# Supplementary material for: Polyacrylonitrile-b-Polystyrene Block Copolymer-Derived Hierarchical Porous Carbon Materials for Supercapacitor
Source: Polymers (Basel). 2022 Nov 24;14(23):5109. doi: 10.3390/polym14235109 (PMC9739205; doi:10.3390/polym14235109)
Supplement: Supplementary file 1 [file polymers-14-05109-s001.zip › polymers-2024485 supplementary update.pdf]

Supporting information

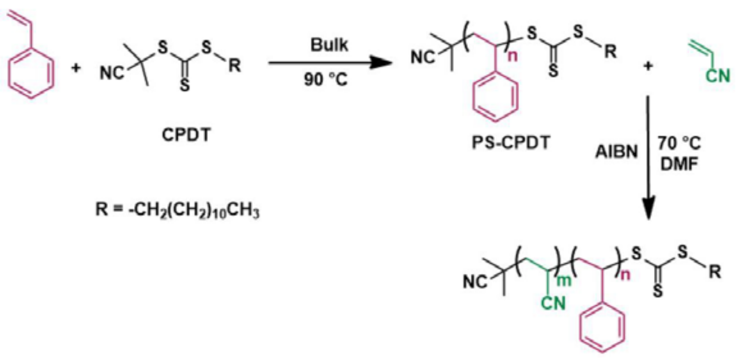

Scheme S1. Synthetic route *via* RAFT polymerization for the obtention of PS-*b*-PAN block copolymer.

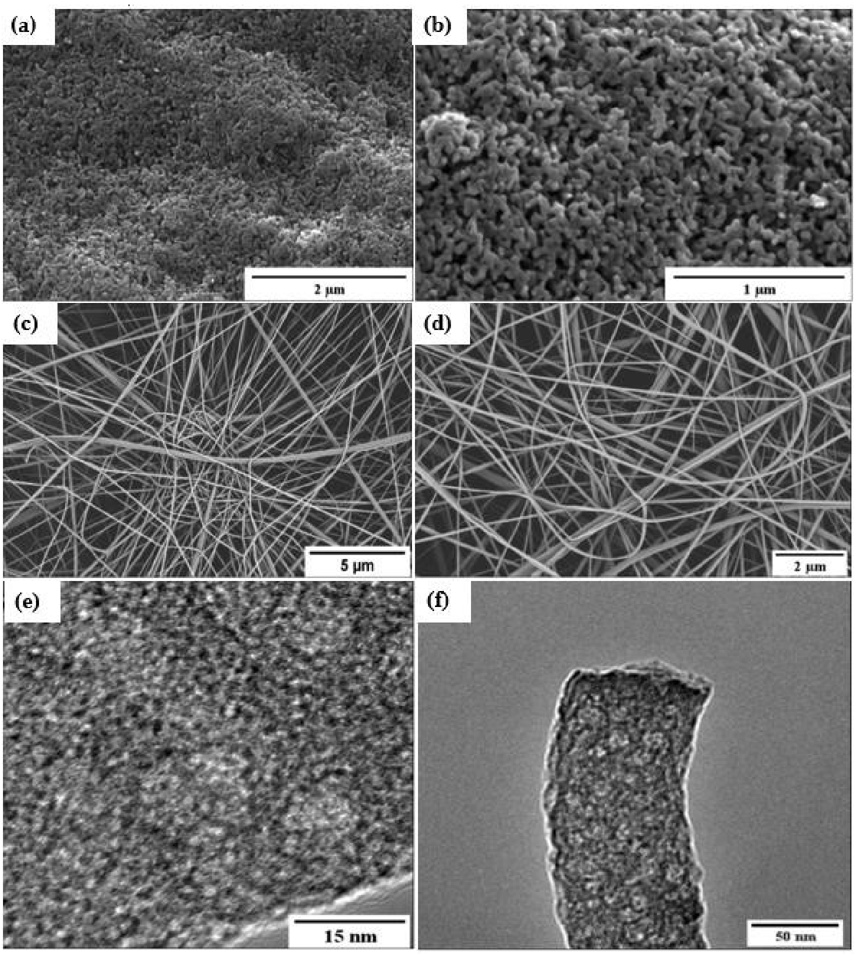

Figure S1. (a) and **(b)** Supplemental SEM images of bulk material after carbonization, **(c)** and **(d)** electrospun PS-*b*-PAN fibers, **(e)** and **(f)** TEM images of the porous carbon fiber.

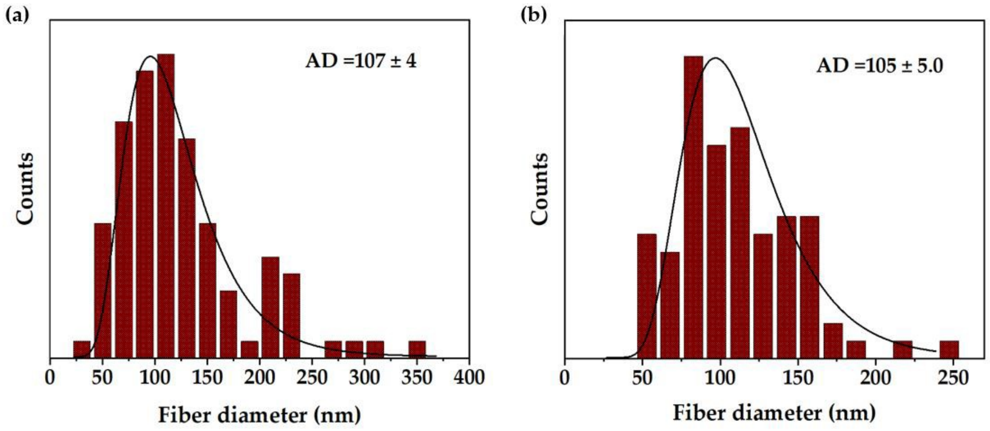

Figure S2. Diameter distribution of fibers **(a)** before and **(b)** after carbonization.

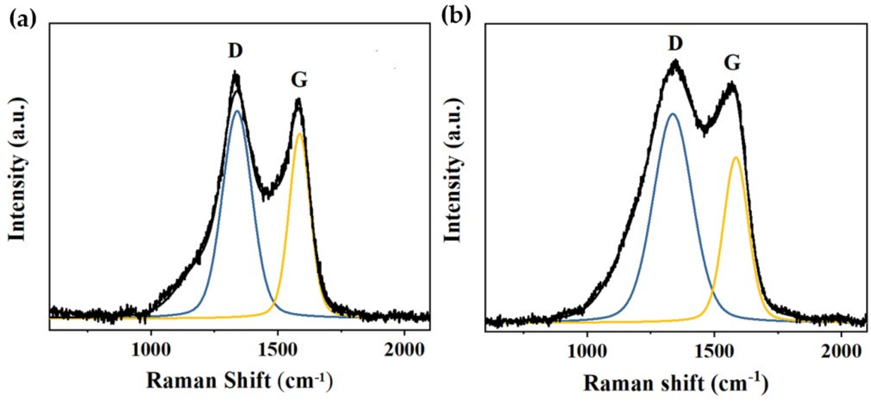

Figure S3. Deconvolution of RAMAN spectra of **(a)** fiber and **(b)** bulk material.

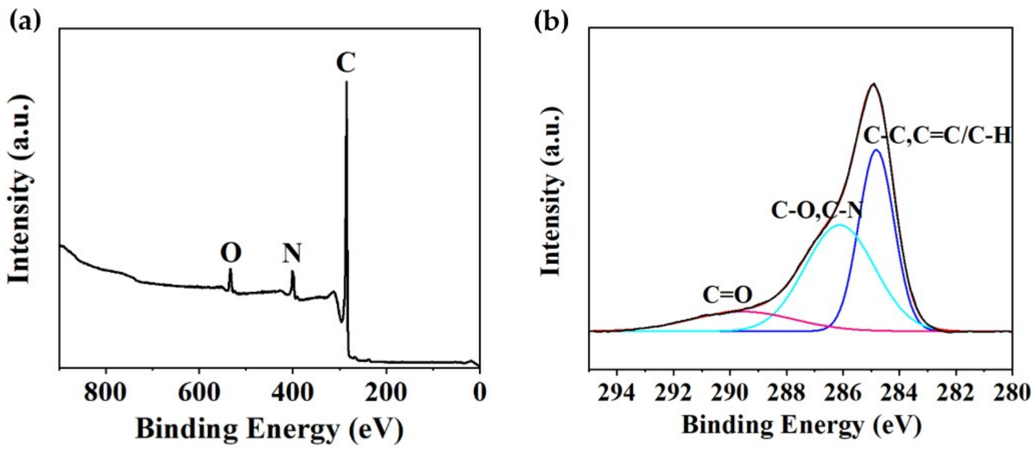

Figure S4. **(a)** XPS full spectrum and **(b)** High-resolution XPS spectra showing C1s peaks in XPS of carbon porous fiber.

Table S1. Relative surface contents of nitrogen and oxygen species determined from N1s and O1s peaks in XPS spectra.

Specie	Content (%)
N	7.37
O	4.57
C	88.06
N-P	36.14
N-C=O	10.39
N-X	45.64
N-O	7.79
O-I	7.88
O-II	36.13
O-III	26.93
O-IIII	27.88

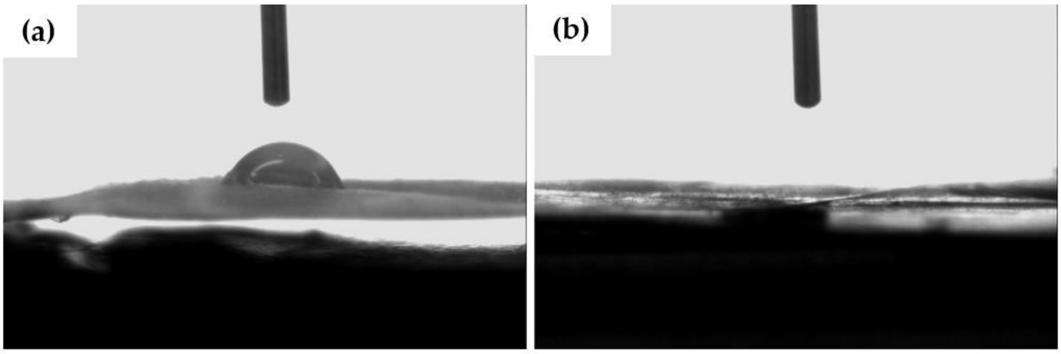

Figure S5. Contact angle of the fiber mat (a) before and (b) after carbonization.

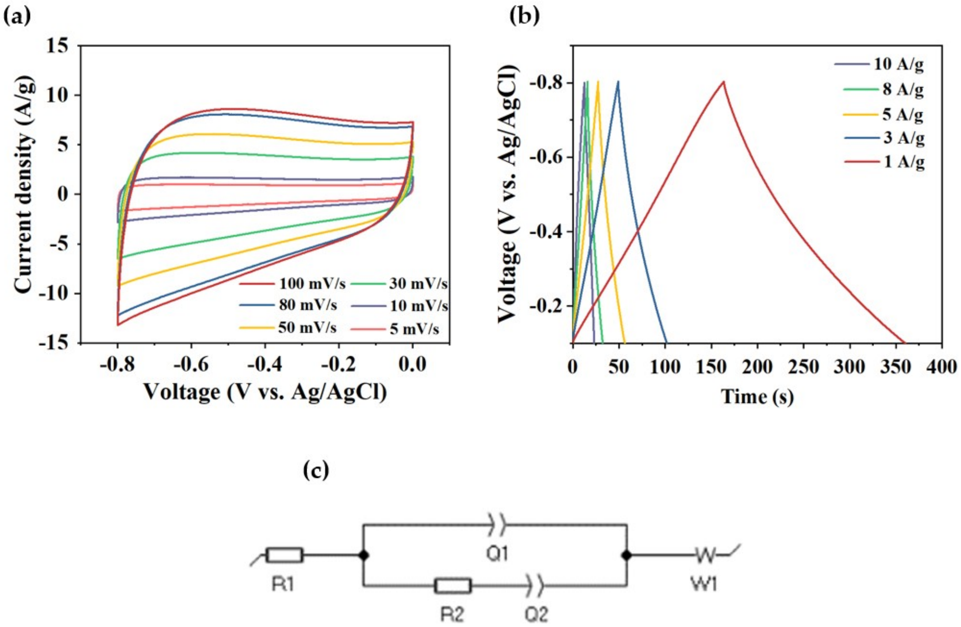

Figure S6. (a) CVs of the fiber electrode at scan rates of 5–100 mV s^{-1}, (b) GCD curves at different current densities of the carbon fiber electrode, and (c) Equivalent circuit used for the EIS fitting data.
